# Effects of Hyperoxia on Mitochondrial Homeostasis: Are Mitochondria the Hub for Bronchopulmonary Dysplasia?

**DOI:** 10.3389/fcell.2021.642717

**Published:** 2021-04-30

**Authors:** Yu Xuefei, Zhao Xinyi, Cai Qing, Zhang Dan, Liu Ziyun, Zheng Hejuan, Xue Xindong, Fu Jianhua

**Affiliations:** Department of Pediatrics, Shengjing Hospital of China Medical University, Shenyang City, China

**Keywords:** mitochondria, hyperoxia, lung development, bronchopulmonary dysplasia, alveolarisation

## Abstract

Mitochondria are involved in energy metabolism and redox reactions in the cell. Emerging data indicate that mitochondria play an essential role in physiological and pathological processes of neonatal lung development. Mitochondrial damage due to exposure to high concentrations of oxygen is an indeed important factor for simplification of lung structure and development of bronchopulmonary dysplasia (BPD), as reported in humans and rodent models. Here, we comprehensively review research that have determined the effects of oxygen environment on alveolar development and morphology, summarize changes in mitochondria under high oxygen concentrations, and discuss several mitochondrial mechanisms that may affect cell plasticity and their effects on BPD. Thus, the pathophysiological effects of mitochondria may provide insights into targeted mitochondrial and BPD therapy.

## Introduction

Morphological findings have shown that preterm infants are born with immature lungs in the saccular stage or even the canalicular stage (Joshi and Kotecha, 2007; Rodriguez-Castillo et al., 2018). Similarly, postpartum lung tissues in rodent models are far from mature and require postpartum differentiation and alveolar regeneration (Silva et al., 2015). Immature alveolar epithelium becomes the first line of exposure to the external environment, and if exposed to oxygen over the long term, lung development is simplified that ultimately leads to bronchopulmonary dysplasia (BPD) (Massaro et al., 1975; Dasgupta et al., 2020). A series of adverse factors, such as long-term postpartum period or inappropriate hyperoxic treatment, mechanical ventilation, infections, and chemical stimulations, can cause an imbalance between alveolar damage and pre and postnatal repair and damage to the immature lungs, resulting in developmental arrest of the alveoli and pulmonary blood vessels (Jensen et al., 2019; Bonadies et al., 2020).

The pathological changes associated with BPD present a simplified model of alveolar and pulmonary vascular structure that is now referred to as “new BPD” (Collins et al., 2017; Jensen et al., 2019). Studies have confirmed that exposure to high oxygen concentrations can cause mitochondrial damage, simplify the lung structure, and ultimately lead to BPD (Ratner et al., 2009; Narala et al., 2018). Effective oxidative metabolism and low reactive oxygen species (ROS) levels are essential for stem cell self-renewal and preserving homeostasis. During embryonic development, mitochondria regulate organ differentiation and development (Lima et al., 2018), cellular energy production, redox reactions, aerobic glycolysis, calcium signaling, ROS generation, macromolecule synthesis, and other biological activities (Smith and Gallo, 2018).

The process of lung development begins at the embryonic stage. Lung buds from the ventral endoderm of the foregut continuously branch along the proximal–distal axis through ordered tissue events (Joshi and Kotecha, 2007; Metzger et al., 2008). Development gradually progresses through epithelial–mesenchymal interactions with the subsequent involvement of several transcription and growth factors, such as the epidermal (EGF), vascular endothelial (VEGF), and fibroblast (FGF) growth factors, and bone morphogenetic protein (BMP), resulting in alveolarization and lung development (Demayo et al., 2002; Serls et al., 2005; Lignelli et al., 2019). The lung tissue of premature infants is relatively hypoxic in the fetal environment for two major reasons: (1) the amniotic fluid contains <1% oxygen (Sjostedt et al., 1958), and (2) fetal blood is ∼40% normative because of pulmonary and cardiac shunts. This seems to favor differentiation and development of the lung at a later stage as a part of preparing the organ for the postnatal demands of gas exchange. The premature lung under hyperoxic conditions is prone to high oxidative stress that can lead to mitochondrial damage owing to the less developed antioxidant capacity of the lung. This review primarily focuses on the pathological processes of late stage lung development under exposure to hyperoxia, discusses the role of mitochondria in alveolar dysplasia, and describes the effects of mitochondrial function on potential pathophysiological effects during epithelial cell development. In addition, we have updated the current understanding of the application of preclinical and clinical mitochondria-targeted therapies for BPD.

## Morphological Abnormalities in Mitochondria

Mitochondria are organelles with an outer (OMM) and an inner (IMM) membrane, an intermembrane space (IMS), a matrix, and they have their own DNA and protein structures (Gail and Lenfant, 1983). The OMM is primarily associated with protein exchange; folded cristae of the IMM have proteins associated with oxidative phosphorylation (OXPHOS), including the mitochondrial electron transport chain (ETC) proteins and ATP synthase, distributed over them (Aravamudan et al., 2013; Prakash et al., 2017; Zhang et al., 2017). More than 40 cell types with different mitochondrial densities exist in the lung tissue; for example, the numbers of mitochondria can be up to 31-fold higher in alveolar type 2 (AT2) than in type 1 (AT1) cells (Massaro et al., 1975). Mitochondria can participate in alveolar epithelial cell activities through complex functions and mechanisms. The number and function of mitochondria continue to increase during postnatal growth and development (El-Merhie et al., 2017). Postnatal mitochondrial changes in AT2, ciliated, and club cells at different stages have been assessed by transmission electron microscopy (TEM) in neonatal mice. Starting on day 15, the mitochondrial structure of AT2 gradually expands and elongates, and the cristae gradually become denser. As glycogen deposits in the cells decrease, the number of lamellar bodies also gradually increases. The mitochondrial fragmentation index of pulmonary vascular endothelial cells increases from 6 h of hyperoxia, and during this time, pulmonary vascular endothelial cells separate from the basement membrane (while the basement membrane remains intact). The ultrastructure of endothelial cell mitochondria is destroyed, and TEM has revealed mitochondrial swelling, vacuolization, disrupted mitochondrial structure, matrix shrinkage, wider and fewer cristae, and disorganization (Ma et al., 2018). The endoplasmic reticulum of endothelial cells is swollen with wider gaps (Teng et al., 2017). However, after 72 h of hyperoxic exposure, the phospholipid content in mouse lung epithelial cells significantly increases (Lee et al., 2013). These results suggest that exposing premature infants to oxygen can disrupt the structure of mitochondria and other organelles in lung cells, and the level of damage gradually increases with prolonged exposure.

## Cell and Mitochondrial Plasticity

**FIGURE 1:**
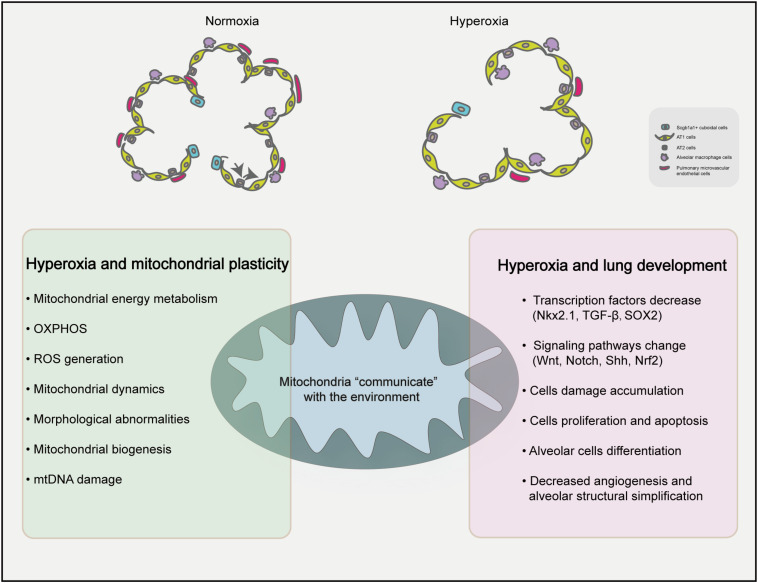
Proposed mechanisms of mitochondrial homeostasis and lung development under hyperxia. Hyperoxia exposure can simplify the lung structure of premature infants, reduce the number of alveoli and reduce the number of microvascular in the lungs compared with the normal group. High oxygen can affect the plasticity of mitochondria and influence BPD.

The basis of alveolus formation is in the immature inter-airspace wall, which consists of two capillary layers and a central sheet of connective tissue (Burri, 2006). The number of alveoli increased significantly increases after delivery, and with the continuous progression of alveoli, the number of alveoli increased by 17.1 times (Burri, 2006). It is worth noting that mitochondria are highly plastic organelles (Bahat and Gross, 2019) and play crucial role in stem cell biology through ROS generation, TCA cycle metabolite production, NAD^+^/NADH ratio regulation, pyruvate metabolism and mitochondrial dynamics (Tan and Suda, 2018). Transcription factors (Nkx2.1, SOX2, Nrf2) and signaling pathways (Wnt pathway, Notch pathway and Shh pathway) related to lung development can be regulated by mitochondrial plasticity (Khacho et al., 2016). AT2 cells are strictly regulated to inhibit the amount of proliferation under baseline conditions and to rapidly and restore barrier integrity after acute injury. Yee et al. (2016) detected alveolar epithelial cell-specific markers during postpartum exposure to different concentrations of oxygen (12, 17, 21, 40, 60, or 100%) and found that the AT2-specific markers pulmonary surfactant-associated protein C (Sftpc) and ATP-binding cassette sub-family A member 3 (Abca3) had a nonlinear response to oxygen during the postpartum expansion of the alveolar epithelium, however, EGFP labeled AT2 don’t produce labeled AT1 cells in neonatal mice. These findings suggest that oxygen in the environment increases AT2 self-renewal ability. Scgb1a1^+^ cuboidal cells in the terminal bronchi are reprogrammed and differentiated under the influence of niches formed in the surrounding environment and dynamic changes in intracellular signals (Parekh et al., 2020). Figure 1 summarizes the contents of this paragraph, changes in the environment affect the oxygen dialogue mitochondria and the environment, which may affect the process of lung development. The plasticity and adaptability of alveolar epithelial cells under changing conditions enables lung epithelial cells to maintain the homeostasis and equilibrium of self-renewal through signal circuits (Demayo et al., 2002). Imbalanced mitochondrial plasticity, however, may be responsible for stunted lung development.

## Oxygen Sensing by Mitochondria

Although the mechanism by which mitochondria use oxygen sensing is of pivotal significance, theories on postpartum alveolar oxygen sensing are scant. The human body contains chemoreceptors such as the carotid sinus and the aortic body that monitor the internal oxygen concentration by sensing changes in the vicinity. However, the molecular responses of living cells to altered gas conditions have been difficult to determine. Nevertheless, the continuous emergence of new techniques and technologies has facilitated more investigative options (Tretter et al., 2020). At the cellular level, 80 and 20% of available oxygen is utilized by mitochondria and other organelles, respectively. As the main oxygen consumer and metabolizer, mitochondria normally function under a partial pressure of oxygen of only 1–3 mmHg (Loenarz, 2020). According to Henry’s law, the oxygen exchange capacity of lung epithelial cells is associated with the intracellular partial pressure of oxygen, the thickness of the cell membrane, and the density of erythrocytes (Weibel, 2017). At high oxygen concentrations, proteins other than hemoglobin gain oxygen-carrying capacity, which increases the ability of oxygen to diffuse from plasma into the mitochondria of various cells (Echevarria et al., 2007).

The way by which cells sense and respond to changes in oxygen levels through hypoxia-inducible factor-1 (HIF-1) has been determined (Wang and Semenza, 1993). When the oxygen supply is sufficient for a cell to meet its energy demand, HIF hydroxylase inhibits HIF through the ubiquitin–proteosome degradation pathway. Conversely, HIF is induced under hypoxia and can activate subsequent target genes, including those that regulate angiogenesis, metabolism, and erythropoiesis, leading to increased blood and oxygen supply to hypoxic tissues (Webb et al., 2009). Current studies have focused more on mitochondrial oxygen sensing and signal transduction under hypoxia, rather than hyperoxia. However, fluctuations in oxygen levels caused by intermittent positive pressure ventilation triggers cells to exit a hyperoxic non-hypoxic state that produces a reaction similar to hypoxia, called the “hyperoxia–hypoxia paradox” (Hadanny and Efrati, 2020). Therefore, the primary mechanism mediating changes in the alveolar epithelium under hyperoxia conditions is believed to increase AT2 oxygen-uptake capacity. Based on this theory, hyperoxia exposed-premature infants and animal models might be exposed to fluctuating, and relatively low oxygen levels (Wohlrab et al., 2021). Another classical theory describes that increased O_2_ content triggers an imbalance of ROS production by intracellular oxidase/antioxidant enzymes, increasing ROS production, and unbalancing the ETC, which leads to the conversion of mitochondrial energy and regeneration of tissue adaptation. Excessive ROS levels are not direct agents of damage but serve as signaling molecules that affect normal cellular function and cause damage to mitochondrial DNA (mtDNA) (Schumacker et al., 2014).

## Oxidative Stress in Mitochondria

Several studies have investigated mitochondrial damage caused by cellular ROS that comprise a primary cause of BPD in pediatric patients (Valencia et al., 2018; Wang and Dong, 2018). Mitochondria are important sites where cells produce ROS (Collins et al., 2017). Compared to those in adult lung, neonatal lung mitochondria produce higher amounts of ROS, whereas cytoplasmic ROS levels are relatively low (Berkelhamer et al., 2013). Neonatal animals can also tolerate hyperoxia better than adults (Koo et al., 2005). However, preterm human infants with non-fatal oxidative stress damage after delivery can develop impaired mitochondrial function, which in turn affects cell maturation and tissue development, ultimately leading to brain damage and BPD (Ten, 2017). The effects of hyperoxia on immature lungs are regulated by development, and the duration of hyperoxic exposure is associated with ROS levels and the degree of pulmonary vascular dysplasia. Neonatal mice exposed to 75% oxygen for 72 h had reduced alveoli and septa, increased vascularization of resistant pulmonary arteries, and right ventricular hypertrophy (RVH) compared with normoxic controls at early, but not later stages (Datta et al., 2015).

Endogenous oxygen free radicals are produced primarily by the mitochondrial respiratory chain, NADPH oxidase (NOX), xanthine oxidase (XO), and other oxidative enzymatic pathways. Hyperoxic conditions can damage alveolar epithelial cells through NOX, and increase elastin deposition in the alveolar septum (Auten et al., 2009). The expression of NOX2 and NOX4 in vascular endothelial cells increases under hyperoxia (Pendyala et al., 2009) and can activate two antioxidant response elements (ARE) on the NOX4 promoter in pulmonary vascular endothelial cells. Loss or mutation of nuclear factor erythroid 2-related factor 2 (*Nrf2*) can cause mitochondrial dysfunction and metabolic disorders induced by ROS (Cho and Kleeberger, 2020). The transcription level of NOX4 can also promote angiogenesis and migration through Nrf2-ARE (Pendyala et al., 2011).

Innovative mitochondrially targeyed triphenylphosphonium antioxidants, such as mitoTEMPO and MitoQ, can scavenge ROS (Oyewole and Birch-Machin, 2015). Specifically, mitoTEMPO targets mitochondria and can prevent hyperoxia-induced lung injury by reducing NOX1 levels, suggesting that the NOX1 production is ROS-dependent (Datta et al., 2015). Classical antioxidants, such as Cu-ZnSOD, MnSOD, and GSH, have been used clinically, but their application as therapeutics has been limited by biochemical and physiological issues (Asikainen and White, 2004). Other antioxidants such as cationic plastoquinone derivatives (SkQ), carotenoids (especially astaxanthin), vitamin E, coenzyme Q10, and resveratrol can also reduce oxidative damage in the mitochondria, but further corroborating studies are required (Teixeira et al., 2018). Further investigation of mitochondrial targeted antioxidants can provide insights into treatment of oxidative stress injury in premature infants.

## Mitochondrial Energy Metabolism

The principal biological function of mitochondria is to produce ATP and deliver energy to cells. Mitochondrial oxidation includes the nicotinamide adenine dinucleotide (NAD)-mediated complex I pathway and the flavin adenine dinucleotide (FAD)-mediated complex II pathway. Reduced NAD (NADH) and reduced FAD (FADH2) are the principal substrates for the redox reactions that facilitate electron transfer and OXPHOS through enzyme complexes I and II (Guo et al., 2018). A comparison of the respiratory chain complex I–IV in AT2 and club cells between 15-day-old and adult mice using PCR and western blotting showed that except for succinate dehydrogenase (SDH) complex II, all other complexes increased gradually over time, suggesting that changes in the morphology and number of mitochondria throughout postnatal development are associated with a higher cellular metabolic demand (El-Merhie et al., 2017).

Regarding the strategy of enhancing oxidative metabolism in lung epithelial and endothelial cells, reactivating metabolic enzymes that protect the integrity of the mitochondrial respiratory chain and matrix membrane during mechanical ventilation and hyperoxia might protect against vascular remodeling (Ten and Ratner, 2020). The tricarboxylic acid cycle is inhibited under hyperoxia, and activities of the key enzyme, that is aconitase (Aco), are decreased (Gardner et al., 1994). In mice, mitochondrial oxygen consumption and ATP production rates are decreased, the surface area of alveoli is reduced, and the numbers of alveoli are significantly reduced under hyperoxia, suggesting that inhibited mitochondrial OXPHOS is associated with bronchoalveolar growth arrest (Ratner et al., 2009). A549 lung epithelial cells decrease mitochondrial spare respiratory capacity at 12 h under hyperoxia and basal respiratory capacity in 48 h. Damage to the mitochondrial respiratory chain under hyperoxic conditions has been analyzed using specific inhibitors of respiratory chain complexes I–V. This is accompanied by an increase in the conversion of glucose to nucleic acid ribose *via* the pentose phosphate pathway (Tetri et al., 2018).

Notably, AT2 cells can secrete special lung surfactants to stabilize the breathing process of the alveoli. Lipid synthesis at the lung surface requires the participation of mitochondria. Hyperoxia affects the fatty acid oxidase, long-chain acyl-CoA dehydrogenase (LCAD), by regulating the mitochondrial lipid synthesis and metabolism pathway, which in turn affects lung surface tension and compliance (Otsubo et al., 2015). This shows that enhanced fatty acid usage helps endothelial cells to maintain their proliferation and alveolarization (Dennery et al., 2018; Yao et al., 2019). The carnitine palmitoyltransferase 1a gene (*Cpt1a*) plays an important role in BPD and fatty acid oxidation prevents hyperoxia-induced endothelial cell damage in neonatal mouse models (Fanos et al., 2014). Adipose fibroblasts in the lung interstitium provide neutral lipids to type II lung cells for the synthesis of phospholipids, which are surfactants in the lungs of immature fetuses. However, hyperoxia can cause the transdifferentiation of adipose fibroblasts into myofibroblasts, accompanied by increased conversion of glucose to the nucleic acid ribose *via* the pentose phosphate pathway. Hyperoxia decreases lipid synthesis *de novo*, which might explain fibroblast formation and the decrease in alveolar type II cell surfactants (Boros et al., 2002; Zhao et al., 2018).

## Mitochondrial DNA Damage

Hyperoxia damages mtDNA in many types of lung cells (Roper et al., 2004; Kandasamy et al., 2019), and such damage can repeatedly increase cellular OS, senescence, and apoptosis (Dobson et al., 2002; Ruchko et al., 2005; Chan, 2006). Human mtDNA is a double-stranded closed loop (16.5 kb) that encodes 13 proteins associated with mitochondrial function, as well as 22 tRNAs and two rRNAs that play significant roles in cells. Unlike nuclear DNA, mtDNA lacks damage repair enzymes and are closer to the source of ROS production, rendering it vulnerable to structural and functional damages (Aravamudan et al., 2013). The mtDNA of alveolar epithelial cells serves as a type of danger-associated molecular pattern (DAMP), as it is released by damaged cells under mechanical stress and activates local fibroblasts (Yang et al., 2020). In addition to responding to mechanical stress, mtDNA produces signaling molecules that communicate with the neighboring cells to promote fibrosis. Damaged mtDNA can be released through mitochondrial permeability transition pores (mPTP) that induce inflammatory and immune responses (Schumacker et al., 2014). For example, ATP released by damaged alveolar epithelial cells under mechanical pressure activates NLRP3 in the endothelial cells to promote endothelial–mesenchymal transition, interacts with P2X7R in macrophages to induce IL-1B production, and locally activates fibroblasts (Yang et al., 2020).

A study of the mechanism of hyperoxia-induced mtDNA damage found that mtDNA repair mediated by the targeted DNA repair enzyme endonuclease III (EndoIII) decreases ROS production and returns branching morphology and levels of surfactant protein C (SFPTC) mRNA to normal in the lungs of neonatal rats (Gebb et al., 2013). The mitochondrial targeted DNA repair enzymes 8-oxoguanine DNA glycosylase (mt-OGG1) and Aco-2 reduce hyperoxia-induced mtDNA damage, thus reducing the apoptosis of alveolar epithelial cells (Kim et al., 2014).

The targeted repair of mtDNA of pulmonary artery endothelial cells with oxidative damage using OGG1 inhibits apoptosis caused by the activation of caspase-3 *via* the xanthine oxidase pathway (Ruchko et al., 2005). Exposing neonatal C57BL/6 mice to 75% oxygen for 14 days causes increased peroxide production and alveolar simplification, and poorer lung function (increased resistance, decreased compliance) compared with normoxic mice (Dylag et al., 2019; Kandasamy et al., 2019). This is important because it shows that mitochondrially encoded genes confer sensitivity or tolerance to oxygen-induced neonatal respiratory diseases. The above-mentioned studies show that targeted repair of mtDNA can reduce hyperoxia-related damage to lung cells. Survivors of preterm birth have increased risk of neurologic and cardiopulmonary disease as they age (Hwang and Rehan, 2018). Hyperoxia has been used to model the oxidative effects of aging. There are many examples of how mitochondria accumulate DNA damage as people age. The question is whether disorders in survivors of preterm infants are related to mitochondrial damage at birth. Perhaps, the threshold of damage is high in them that eventually leads to development of such metabolic diseases later in life.

## Mitochondrial Biogenesis

Mitochondria are central hubs of catabolic and anabolic reactions that ensure sufficient cellular metabolic adaptation during development and differentiation. At the transcriptional level, mitochondrial biogenesis is regulated by the mitochondrial and nuclear genes (Jornayvaz and Shulman, 2010). Peroxisome proliferator-activated receptor γ coactivator α (PGC-1α) is a master regulator of mitochondrial biogenesis, belonging to a family of transcriptional coactivators that also includes PGC-1β and the peroxisome proliferator-activated receptor gamma, coactivator-related 1 (PPRC1). PGC-1α activates various proteins, including nuclear respiratory factors 1 and 2 (NRF1 and NRF2) and mitochondrial transcription factors A and B (TFAM and TFBM), and regulates the transcription and translation of mtDNA (Jornayvaz and Shulman, 2010). TFAM is critical for the development of the lung. Notably, fetuses harboring a TFAM mutant exhibit branching defects during embryonic development and incomplete peripheral airways; thus, they cannot survive after birth (Srivillibhuthur et al., 2018). Mitochondrial biogenesis can be regulated *via* the AMPK–PGC-1a and SIRT1–PGC-1a axes, which play a central role in promoting mitochondrial energy metabolism and biogenesis (Lehman et al., 2000; Liang and Ward, 2006). Tracking the relationship between mitochondrial biogenesis and acute lung injury, inflammation, and pulmonary fibrosis using mitochondria-specific green fluorescent protein, has revealed that mitochondrial biogenesis is most prominent in pulmonary vascular endothelial cells during hyperoxia-induced lung damage (Carraway et al., 2008).

Under various stimulated environmental factors, hormones, secondary messengers (calcium, endothelial nitric oxide synthase eNOS, cAMP), and kinase pathways (PKA, MAPK, PRKAA2) can regulate the expression and post-translational modification of PGC-1α *via* regulation of protein localization and function. Regulating PGC-1α can affect the transcription of a variety of steroid receptors and nuclear receptors, and mediate the transcriptional activity of PPAR-γ and thyroid hormone receptor on the uncoupling protein promoter (Zhu et al., 2013). It can also regulate the key mitochondrial genes involved in adaptive thermogenesis by coordinating the expression of a large number of genes involved in glucose and fatty acid metabolism. Studies have shown that the clinical drugs used to improve breathing, such as aminophylline and montelukast, can promote the biogenesis of human alveolar epithelium mitochondria through cAMP responsive element binding protein (CREB)/PGC-1α (Wang et al., 2019; Wei et al., 2019). The use of the mitochondrial antioxidant resveratrol can protect mtDNA and reduce apoptosis through SIRT1–PGC-1a (Wang et al., 2020; Zhu et al., 2021). These findings suggest that the biogenesis of mitochondria plays an important role in protecting against hyperoxic lung injuries.

## Mitochondrial Dynamics

Mitochondria are in a dynamic equilibrium of continuous fusion and fission, which plays an important role in energy metabolism and maintaining stability of the intracellular environment, as well as the differentiation and development of cells. Mitochondrial division is mainly mediated by dynamin-related protein 1 (Drp1) and mitochondrial fission protein 1 (Fis1); mitochondrial fusion is mediated by the IMM fusion optic atrophy 1 (OPA1) and mitochondrial fusion by the fusion proteins (mitofusin1 and 2, Mfn1, and Mfn2). The level of oxidative stress can cause imbalance in the levels of the above-mentioned proteins, and thereby resulting in mitochondrial fragmentation (Wu et al., 2011). Some researchers suggest that mitochondrial fusion and fission are unbalanced in lungs damaged by hyperoxia, which directly inhibits their function (Ten and Ratner, 2020; Lee et al., 2020). Reportedly, mitochondrial fragmentation of lung endothelial cells increased, p-Drp1 increased, Mfn1 decreased, OPA1 increased, and the autophagy-related proteins P62, PINK-1, and LC3B increased compared with the control group after 48 h of hyperoxia. However, exposing the cells to air for 24 h could reverse the trend (Ma et al., 2018). Recently, Teng et al. (2017) found that caffeine used to stimulate the respiratory center of premature infants can also improve the endoplasmic reticulum stress caused by hyperoxia, and that the endoplasmic reticulum and mitochondria can interact with each other through the participation of MFN2 and DRP1, inducing mitochondrial division.

Mitochondrial dynamics are also regulated by epigenetics. Sirtuins (SIRT) comprise a family of highly conserved NAD^+^ dependent histone deacetylases that are distributed in different parts of the cell (Ma et al., 2018). Reportedly, their expression is decreased under hyperoxic conditions. SIRT3 can deacetylate OPA1, thus increasing the efficiency of mitochondrial fission (Samant et al., 2014) and SIRT1 can regulate SUMOylation, affect mitochondrial dynamics, and reduce damage (Tan et al., 2018). Mitophagy and mitochondrial synthesis and division jointly participate in the quality control of intracellular mitochondria, which is conducive to mitochondrial homeostasis (Pickles et al., 2018). Mitophagy is mainly mediated by the PINK1/Parkin pathway. In addition, other mitochondrial proteins, such as NIX (also known as BNIPL), BNIP3, and FUNDC1, also mediate mitochondrial autophagy (Wei et al., 2015). We have previously shown that oxygen exposure can affect the expression of mitochondrial autophagy (Yu et al., 2020). In conclusion, mitochondrial fusion and fission are unbalanced in the lungs injured by hyperoxia, which directly affects the quality control of mitochondria.

## Clinical Application of Mitochondrial Therapy

Clinical studies have suggested that the maximum mitochondrial oxygen consumption of umbilical vein endothelial cells can serve as an important predictor of BPD, and that mitochondrial function serves as a predictor of the long-term prognosis and mortality rates of preterm infants with BPD (Kandasamy et al., 2017). The quality of placental mitochondria can be used to assess sensitivity to ROS, but its application to determining pathological processes and prognostic relationships with chronic lung disease requires further investigation (Papa Gobbi et al., 2018).

Glucocorticoids play a major role in the regulation of fetal and postnatal lung development, and changes in glucocorticoids can significantly affect the mitochondrial dynamic regulation in lung development (Du et al., 2009); in ways that can persist into adulthood. Changes in glucocorticoid sensitivity and mitochondrial protein abundance can be used to identify those at the highest risk of developing advanced lung diseases (Gnanalingham et al., 2006). Xanthines (theophylline and caffeine) have been widely applied in neonatology for treating apnea in premature infants. Their mechanism of action is primarily by inhibiting adenosine receptor stimulation in the respiratory center, whereas phosphodiesterase inhibition affects bronchodilation (Reyburn et al., 2012). Caffeine attenuates cyclooxygenase-2 activation induced by hyperoxia and protects mitochondria, thus reducing lung damage due to hyperoxia (Teng et al., 2017).

Mitochondrial replacement therapy is proposed to improve pathological outcomes and resolve pulmonary stagnation. Exogenous supplementation with healthy mitochondria is emerging as a potential therapeutic approach that should be investigated in detail, as it improves the status of mitochondrial damage *via* the endocytosis by damaged cells to offset diseases (Agrawal and Mabalirajan, 2016). The delivery of mitochondria through blood vessels of the pulmonary artery or through the trachea (nebulization) could improve lung ischemia-reperfusion damage in C57 mice (Moskowitzova et al., 2020). The transplanted mitochondria initially increase the amount of intracellular ATP and activate ATP synthesis, they then migrate to the target cells through actin-dependent endocytosis, and release protective cytokines that promote cell growth and proliferation. In addition, normal mtDNA of transplanted mitochondria can also replace damaged mtDNA (Eyre-Walker, 2017). The first clinical study of mitochondrial transplantation therapy at Boston Children’s Hospital had a great impact in the field of medicine. They performed autologous mitochondrial transplantation for myocardial ischemia–reperfusion injury in pediatric patients who required extracorporeal membrane oxygenation (ECMO). Following transplantation, most of the dysregulated cardiac functions were recovered in the patients and they were successfully freed from ECMO support (Emani et al., 2017). However, there are several issues that need to be overcome for improving the efficiency and success of mitochondrial transplantation therapy. For example, a single administration of mitochondria does not result in the maintenance of long-term therapeutic efficacy. In the above-mentioned case, the method of mitochondrial isolation, mitochondrial source, route of administration, and number of doses were dependent on the ease of performing mitochondrial transplantation. Therefore, optimal standard protocols for targeted mitochondrial transplantation therapy need to be developed (Yamada et al., 2020). Since mitochondria in the extracellular space can also serve as DAMP that activate inflammation and exacerbate damage, clinical mitochondrial transplantation requires considerable caution that cannot be overlooked (Zhang et al., 2010).

## Discussion

**FIGURE 2:**
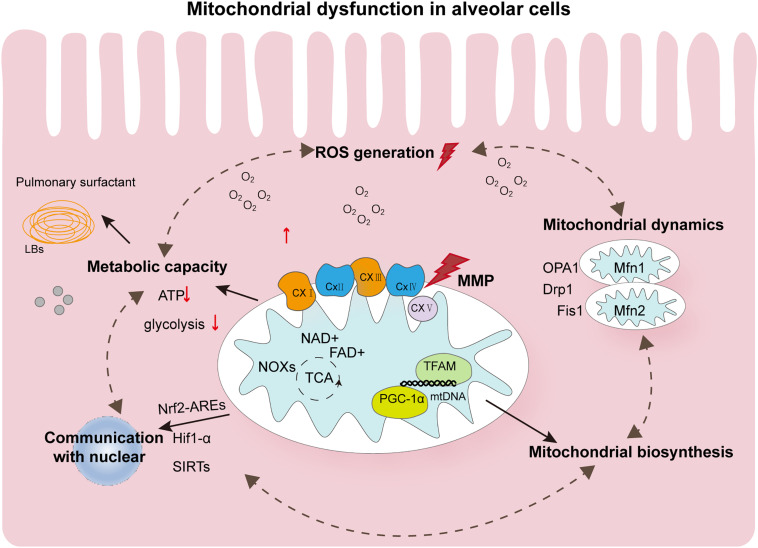
Summary of the major pathophysiological events contributing to mitochondrial dysfunction in hyperoxia-exposed neonatal lung. Oxygen affects the mechanism of alveolar morphology mainly through mitochondrial dysfunction. Altered ROS levels and mitochondrial metabolism in alveolar cells and mitochondrial homeostasis disorders are involved in the occurrence of BPD. These states of dysfunction are interdependent and can further form loops that influence each other. They can also be simultaneously affected by controlling the quality of mitochondria. In addition, mitochondria in alveolar cells communicate with the nucleus by regulating several transcription factors, such as Nrf2-AREs, HIF-1α, and SIRTs, and undergo epigenetic modifications. All these aspects provide possible evidence for the important role of mitochondria in BPD. ROS, reactive oxygen species; AT2, alveolar type 2; BPD, bronchopulmonary dysplasia; LBs, Lamellar corpuscle; ATP, adenosine triphosphate; OPA1, optic atrophy 1; Drp1, dynamin-related protein 1; Fis1, mitochondrial fission protein 1; Mfn1, mitofusins protein 1; NOX, NADPH oxidase; TFAM, mitochondrial transcription factor A; TFBM, mitochondrial transcription factor B; PGC-1α, peroxisome proliferator-activated receptor γ coactivator 1-α.

The role of mitochondria in postnatal lung development remains poorly understood with respect to the mechanisms that cause alveolar cavity enlargement and structural simplification during oxygen therapy for lung damage caused by lack of oxygen. The pathophysiological mechanism of mitochondrial damage in BPD is extremely complex. In this article, we summarize that hyperoxia will affect the homeostasis of mitochondria. These dysfunction states are interdependent and will affect each other and thus affect developmental results, as shown in Figure 2. As a consensus view of neonatologists, hyperoxia inhibits angiogenesis, stimulate expansion of alveolar epithelial cells, and promotes recruitment of inflammatory cells into the lung. However, each cell type responds differently to hyperoxia. Despite extensive research, the way by which different cell types respond to hyperoxia remains poorly understood that warrants further investigation. Mitochondrial damage in BPD is associated with changes in signal transduction and energy metabolism. How the quantity and function of mitochondria affect hyperoxia-exposed lungs at the tissue and cellular levels is one of the keys to further understanding the pathogenesis of hyperoxia-induced mitochondrial damage. Further, in-depth studies are required to elucidate how the dynamic changes in mitochondrial functions affect the outcomes of lung development in preterm infants during the repair of reversible damage caused by hyperoxic lung injury. A clearer understanding of the mechanisms of mitochondrial damage and its associated upstream and downstream regulatory pathways will deepen our understanding of the pathological processes of BPD, which holds major implications in appropriate oxygen therapy as well as the diagnosis and treatment of preterm infants with BPD.

## Author Contributions

YX wrote the manuscript and designed the figures. ZX, CQ, ZD, LZY, ZH, XX, and FJ wrote specific parts of the manuscript and reviewed the final draft of the manuscript. All authors contributed to the article and approved the submitted version.

## Conflict of Interest

The authors declare that the research was conducted in the absence of any commercial or financial relationships that could be construed as a potential conflict of interest.
